# A Workable System of Cottage Hospital Book-Keeping

**Published:** 1914-04-11

**Authors:** 


### DOMESTIC CONTROL IN LITTLE HOSPITALS.

### A Workable System of Cottage Hospital Book-Keeping.

In many small institutions there is lack of control on account of the scarcity of labour. There is, of course, no paid secretary, and the honorary secretary is usually a busy man who can only spare an hour or two occasionally to check the accounts and ensure that all vouchers, etc., are correct. The accounts depend on the business faculty of the matron, who is often handicapped by finding an antiquated, slipshod system in use, and is content to concentrate her efforts on accounting rigidly for every penny which is spent. In cottage hospitals and convalescent homes the number of inmates varies largely from week to week. It is rare to find that any note is made of the numbers boarded in the establishment, and estimates of the cost of the household are largely a matter of guesswork.

It is not possible to introduce a complicated system of accounts into such households. Even if an elaborate set of books were started it is hardly probable that in the absence of any clerical assistance the matron could keep them up. Yet the hinge on which economy turns is knowledge of what the household costs per head. How can this be ascertained all the year round without worrying the staff?

We discovered not long ago a very simple, workable system in use at the Cottage Hospital at Lyme Regis, which was thought out and introduced by one of the honorary officers. The hospital is a small one, and was built in the Jubilee year 1887 to serve the needs of a widely scattered population. It contains eight beds in all, the daily average occupied is three, and the patients for the most part contribute towards their maintenance from 3s. 6d. to 5s. a week. There are one or two beds in a private ward for which a higher charge is made. The staff consists of matron, assistant nurse, and probationer, with a general servant. A certain amount of district work is undertaken by the matron and her assistant, involving no less than 495 visits during the past year, so that it will be readily understood that there is little time available for what may be called book work.

The form (A.) given below, which we have slightly modified, is in use for noting the inmates present in the hospital throughout the month, with the weekly sum contributed by each.

To fill in this form day by day is a matter of a minute or two, yet it is the basis on which control over domestic matters is built. The number of patients varies from eight, or in emergencies nine, to *nil*. It will readily be seen that the bills must undergo great fluctuations according to the numbers in residence, and by keeping an exact account of the numbers boarded day by day the matron enables those responsible for the finance to keep their finger on the pulse, as it were, of the household.

### Monthly Bills.

It will be seen that the form is drawn up for a month's use, and we think that in small and very busy establishments there is much to be said for the practice of making the unit of cost the month instead of the week. A weekly calculation of cost imposes unnecessary strain on both matron and hon. secretary. It is rarely that time can be systematically set apart on the same day of the week for the necessary scrutiny. This leads to slackness or perfunctory approval of unexamined accounts on the part of the honorary officer, while the matron is exposed to the worry of constantly preparing statements which are not used when made. It saves much trouble if the tradesmen's books are made up to the end of the month and paid monthly. They must, of course, be sent in every week for examination; every item should then be checked by the matron, and her initials placed at the end.

### Household Books.

But by no means the larger part of the expenditure will pass through the hands of the regular tradesmen. In small country institutions great economies can be effected if the matron pays for produce at the door. Butter, eggs, poultry, fruit, vegetables, and other articles are brought in, sometimes from great distances, by the country people,

*Form A.* *Showing Inmates in Hospital, with weekly sum contributed by each.*

| Patients' Names | Days of the Month | | | | | | | | | | | | Total Days Resident | Amount Charged | Weekly Payment | | | | |
|---|---|---|---|---|---|---|---|---|---|---|---|---|---|---|---|---|---|---|---|
| | 1 | 2 | 3 | 4 | 5 | 6 | 7 | 8 | 9 | | 30 | 31 | | | 1 | 2 | 3 | 4 | Total |
| | | | | | | | | | | | | | | s. d. | s. d. | s. d. | s. d. | s. d. | |
| A ... ... ... | 1 | 1 | 1 | 1 | | | | | | | | | 14 | 3 6 | 3 6 | — | — | — | — |
| B ... ... .. | | | 1 | 1 | 1 | 1 | 1 | 1 | 1 | ... | | | 19 | 5 0 | 5 0 | 5 0 | 5 0 | | |
| C ... ... ... | | | | | 1 | 1 | 1 | 1 | 1 | ... | 1 | 1 | 15 | 3 6 | | | 3 6 | 3 6 | |
| D ... ... ... | | | | | 1 | 1 | 1 | 1 | 1 | ... | | | 12 | nil | — | — | — | — | |
| E ... . ... | | | | | | | 1 | 1 | 1 | ... | 1 | 1 | 16 | 3 6 | | | 3 6 | 3 6 | |
| F (private) ... ... | | | | | | | | | 1 | ... | | | 14 | 25 0 | | £1 5 | £1 5 | | |
| G ... ... ... | | | | | | | | | | ... | | | | | | | | | |
| Total ... ... | | | | | | | | | | | | | 90 | | 8 6 | £1 10 | £1 17 | 7 0 | |

and can be had in this way direct from the farms cheaper and fresher than they can be purchased at the shops. Hence the matron must have at her disposal a sum of petty cash, so that she can pay ready money for such produce. She will have books for such as supply her regularly, and must obtain signed vouchers from casual vendors. In scrutinising the accounts the honorary officer will then have a double set to examine: those which he pays by cheque at the end of the month and those which the matron presents for articles paid at the time of purchase. In making out the statement for his examination the matron uses the following form:—

*Form B.* *Household Expenses.* *Month............*

| — | Tradesmen's Books | | | Household Book Petty Cash | | | Total | | |
|---|---|---|---|---|---|---|---|---|---|
| | £ | s. | d. | £ | s. | d. | £ | s. | d. |
| Baker ... ... ... ... | | | | | | | | | |
| Milkman ... ... ... | | | | | | | | | |
| Grocer... ... ... ... | | | | | | | | | |
| Greengrocer ... — ... | | | | | | | | | |
| Butcher ... ... ... | | | | | | | | | |
| Etc., etc. ... ... ... | | | | | | | | | |
| Total ... ... ... | 3 | 0 | 0 | 5 | 0 | 0 | 8 | 0 | 0 |

| | No. of Days in Residence |
|---|---|
| Matron ... ... ... ... ... | 31 |
| Assistant ... ... ... ... | 26 |
| Probationer ... ... ... ... | 31 |
| Servant ... ... ... ... ... | 30 |
| Patients (from Form A) ... ... | 90 |
| Total of days ... ... ... | 208 |

£8÷208=9.2*d.*
Average cost of food per head per day, $9\frac{1}{5}$*d.*

The approximate totals for one month have been filled up in the above specimen, though the actual items are left blank. Accompanying the statement of the cost for baker, butcher, grocer, etc., is a statement of the number of days' board supplied during the month, calculated on the number of patients derived from Form A and the number of staff resident during the month. In a small household the comings and goings of any of the staff are too definitely remembered to require daily checking. It suffices if the matron fills in the total days in residence at the end of the month for each member of the staff, deducting any occasion on which they may happen to have been absent. Having this form before him which shows the total cost of the food for the month, extracted partly from the tradesmen's books and partly from the matron's day books of cash purchases, the honorary secretary has merely to divide this sum by the number of days' board, and he obtains the average cost per head per day of the inmates of the hospital for that month.

### No Store-room.

It will be seen that no allowance is made in this system for stores purchased and put by. It does not, in fact, make for economy to purchase in bulk in establishments too small to admit of time being spent in measuring and weighing. Such articles as tea and sugar can be bought by the month, and experience speedily shows almost exactly what will be required. Store-room accommodation is commonly very bad, if not non-existent, in small institutions, and it is far better not to attempt to deal with provisions in bulk. Small quantities are more likely to be used with care by untrained servants, and that will be indeed a wonderfully lucky matron who has not often to depend on unskilled labour in the little hospital.

